# Design and characterization of telecentric f-*θ* scanning lenses for broadband terahertz frequency systems

**DOI:** 10.1063/5.0030110

**Published:** 2020-12-11

**Authors:** Zachery B. Harris, Stefan Katletz, Mahmoud E. Khani, Arjun Virk, M. Hassan Arbab

**Affiliations:** 1 Department of Biomedical Engineering, Stony Brook University, Stony Brook, New York 11794, USA; 2 Department of Neurology 1, Kepler University Hospital, 4020 Linz, Austria

## Abstract

Telecentric beam scanning using f-*θ* lenses offers nearly uniform spot size, linear beam displacement, and normal incidence angle over a planar surface. These unique properties allow for the minimization of imaging distortion over a wide field-of-view. In this article, we present a numerical method for designing custom f-*θ* lenses in the THz regime. We fabricated three lenses made from different commonly used polymer materials in the THz optics. We demonstrated their optical performance metrics compared to a conventional plano-convex lens over the broadband 0.3 THz–1 THz range. We find that the f-*θ* lens designed using the optical properties of high-density polyethylene achieved superior performance by maintaining a constant phase over a wide field of view of about 34°. We demonstrate this isophase property by measuring a constant time of arrival of the THz time-domain pulses over a reference mirror with a standard deviation of ∼19 fs, in excellent agreement with simulation predictions. This work will pave the way for the design and implementation of highly precise and fast telecentric imaging systems in the THz frequencies.

## I. INTRODUCTION

**FIG. 1.**
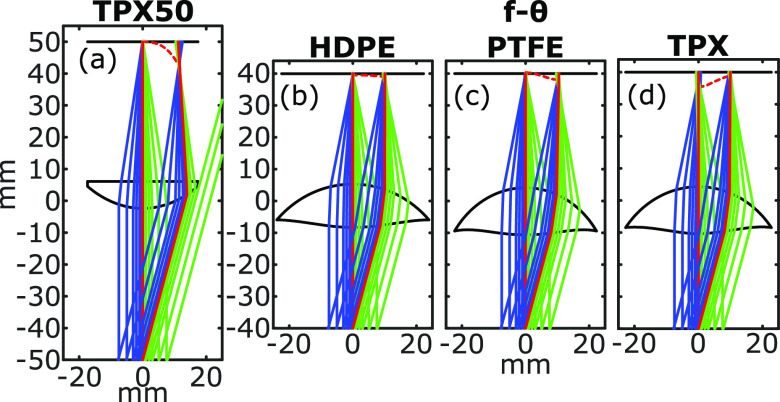
Focusing performance of a commercial plano-convex THz lens, TPX50 (a), is compared to that of f-*θ* lenses designed from HDPE (b), PTFE (c), and TPX (d) for both the chief ray and at *θ* = 15° deflection angles. Red dashed lines show the focal locations.

F-*θ* lenses have found many diverse applications in highly accurate laser beam scanning over the entire electromagnetic spectrum. These applications include drilling precise holes using UV lasers,^1^ optical coherence tomography in the IR band,^2^ and terahertz (THz) imaging,^3–7^ where scanning a planar surface is the objective of the imaging system. As the deflection angle of the collimated beam is increased, the geometrical position of the focal points of a spherical lens, its focal plane, constitutes a curved surface. Therefore, using conventional spherical lenses results in significant image distortions from a planar target. Alternative scanning optics, such as flat-field lenses, have been used to address this problem.^8^ However, they usually result in a non-linear beam displacement pattern on the target surface, which can be modeled and accounted for using a scanning algorithm. In contrast, f-*θ* lenses are designed to provide a linear beam displacement as a function of the deflection angle (*θ*; see Fig. 1), resulting in a constant scan rate on a planar surface. When used in a telecentric system, f-*θ* lenses produce constant spot size and imaging resolution as a function of the deflection angle, thus eliminating distortions at the edges of the field-of-view.^9^ These properties also ensure a broadband THz constant phase over the scanning plane, i.e., the planar focal surface is also an isophase.

While f-*θ* lenses are commonly used in other parts of the optical spectrum from UV to IR, their design, broadband characterization, and the effect of material properties on the lens performance in the THz frequency range have not been widely studied. In this article, we will present a robust numerical method for designing custom f-*θ* scanning lenses in the THz frequencies. We will compare the optical performance of three lenses designed and fabricated using commonly used polymers for manufacturing THz optics, namely, high-density polyethylene (HDPE), polytetrafluoroethylene (PTFE), and polymethylpentene (TPX). Finally, we will demonstrate the temporal performance of the three lenses in excellent agreement with the simulation results using a reflection setup, which uses a 2-axis gimballed mirror (GM) for beam steering. Importantly, we find that the HDPE f-*θ* scanning lens can achieve isophase behavior over a wide 34° field of view, as measured by a constant sub-cycle time of arrival (TOA) ∼1/4 of the pulse duration of the femtosecond probe beam.

## II. LENS DESIGN

Figure 1 shows ray tracing simulations of 15-mm-diameter beams at *θ* = 0° and 15° deflection angles to compare the performance of a conventional plano-convex lens, TPX50 (aspheric, 50 mm focal length, 38.1 mm diameter), to the three f-*θ* lenses (radially symmetric, aspheric, 40 mm focal length, 50.8 mm diameter). The f-*θ* lenses were designed using the optical properties of TPX, PTFE, and HDPE (*n* = 1.46, 1.43, and 1.54, respectively).^10–12^ Exact values of each material’s optical properties vary depending on the manufacturer.^13^ While these polymer materials have relatively similar refractive indices, each produces a slightly different profile. In particular, the higher refractive index of HDPE results in a thinner lens design. Importantly, HDPE also has a negative dispersive property (defined as Δ*n*/Δ*f*), which is three-times and five-times smaller than the positive dispersion of PTFE and TPX, respectively.^12,13^ These simulations show the major advantages of the telecentric f-*θ* designs over the conventional lens. First, they maintain a planar focal surface as the beam angle is steered in a telecentric configuration. Additionally, the focused beam is perpendicular to the target plane over the full deflection range. Therefore, the reflected signal returns by the same path as the incident beam, removing the need for a second set of optics for de-scanning. However, Fig. 1 also shows the variations in the performance behavior between f-*θ* lenses, highlighting the need for full optical characterization of each lens.

**FIG. 2.**
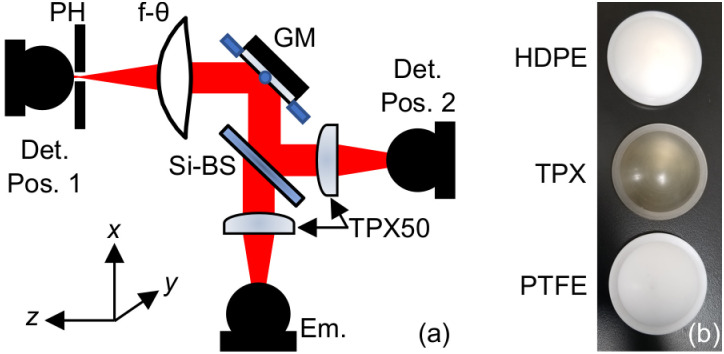
(a) Schematic of the setup used for beam characterization. PH: 1 mm pinhole, GM: gimballed mirror, Si-BS: silicon beam splitter, Em. and Det.: emitter and detector photoconductive antennas, respectively, Det. Pos. 1: the position of the detector for the transmission layout, used for spatial measurements, and Det. Pos. 2: the detector position for the reflection layout, used for alignment and time of arrival measurements. (b) Photographs of the f-*θ* lenses.

Figure 2 describes the experimental setup used to measure the performance of the lenses. The THz emitter and detector were photoconductive antennas (PCAs) as part of the TERA ASOPS time-domain spectrometer (Menlo Systems, Inc. NJ, USA). The 1560 nm fiber-coupled PCA pigtails have integrated silicon lenses designed to be used with the TPX50 lenses for collimation and focusing. The collimated beam passed through a beam splitter before being steered toward the f-*θ* lens by a mirror mounted on a two-axis motorized gimbal (T-OMG, Zaber Technologies, Inc. BC, Canada), used to raster scan the THz beam on the focal plane. The gimballed mirror’s axes of rotation coincided with the front focal point of the f-*θ* lens, thus forming a telecentric configuration. By inserting a pinhole in front of the detector at position 1, we reduced the effective cross-sectional area for a given measurement, allowing for the sampling of smaller portions of the beam. The pinhole and detector were mounted on a motorized 3D stage.

The scanning f-*θ* lenses were designed using MATLAB and a two-dimensional ray tracing code. A bundle of rays was first deflected by the scanning mirror. They then hit the two surfaces of the lens and finally converge on the target plane. The front and back surfaces of the lenses were spherical profiles with polynomial corrections by distance from the optical axis of up to 4th order. Instead of using the radii of the initially bi-spherical lens,^3^ the inverse values were used as fitting parameters, allowing for flat surfaces and a gradual transition from convex to concave solutions. The previous optimization process regularly yielded unrealistically thick lenses. Therefore, we also included a weighted thickness term to account for this effect. The optimization residue is then given by

$$res=w_{t}t+\frac{1}{N_{\alpha }}\underset{\alpha }{\sum }w_{s}s+w_{i}i^{2},$$ (1)

where the summation is over the discrete angles of the scanning mirror, *α*; the spot-size, *s*, is determined as the standard deviation of the positions of the rays on the target plane; and the incidence angle, *i*, is measured from the normal of the incidence plane in degrees. The thickness of the lens, *t*, was measured on the optical axis in mm. The target function was minimized using the Nelder–Mead simplex method.^14^ The isophase behavior is the result of enforcing two conditions in the design equation: planar focus and telecentricity. The former, represented by the spot-size term, guarantees that the bundle of rays converges at a focus at the target plane. This term is equivalent to requiring equal optical path lengths for all rays in a given bundle. The latter, represented by the angle of incidence term, ensures that the central rays of each deflection angle are parallel to the chief axis. Setting the weight parameters to *w*_*s*_ = *w*_*i*_ = 50*w*_*t*_, satisfactory f-*θ* lens profiles were obtained for each polymer. The THz beam stays parallel to the optical axis when scanned over the object plane, and there is a linear relationship between the scan angle of the mirror and position of the focal point (*f* × *θ*). The chosen value for *w*_*t*_ represents a trade-off between the lens thickness and geometrical focusing error, which was well below the diffraction limit for frequencies below the design frequency of 1 THz (corresponding to 300 *μ*m wavelength). For further refinements, previous solutions were used as a starting point and the polynomial coefficients were slightly and randomly varied. A final design was obtained if these iterations resulted in a shape close to the previous optimal solution. As will be seen later, although this perturbation process was intended to ensure that the eventual solutions represented a global residue minimum, in certain cases, a trade-off between optical performance techniques was noted. A 2 mm thick rim was added to the design for mounting, giving each lens a 50.8 mm diameter. The 2D profiles were then revolved to form 3D lens shapes, and the three f-*θ* lenses were fabricated using a CNC (computer numerical control) lathe with a unidirectional positioning accuracy of 0.008 mm/100 mm along the x-axis and 0.010 mm/300 mm along the z-axis and with a repeatability of ±0.002 mm along the x-axis and ±0.003 mm along the z-axis.

## III. LENS CHARACTERIZATION

Each of the three f-*θ* lenses and the TPX50 lens (Menlo Systems, Inc.) were then characterized using multiple performance metrics described below. Ray tracing simulation shows that when the plano-convex TPX50 lens is used in a telecentric imaging scenario, placing it with the flat side facing away from the focal plane, i.e., in reverse to its usual orientation shown in Fig. 1(a), gives a better off-axis focusing performance. Therefore, the reverse orientation was used in the following measurements. Lens alignment was accomplished by taking two perpendicular reflection line scans over a flat mirror placed at the focus and perpendicular to the beam path. For the horizontal and vertical alignment, the lens was placed at approximately its nominal axial location and adjustments were made until the difference in the time of arrival at various beam angles from that of the chief ray was symmetrical about 0° in both axes. Fine tuning of the axial position of the lens was guided by comparing the shape of the time-domain traces to those produced by two-dimensional simulations.

### A. Beam profiles

Beam profiles were measured using the detector in the transmission position of Fig. 2 with the 1 mm diameter pinhole placed at the nominal focal length of the lens. The detector and pinhole were then moved in a raster pattern with 0.5 mm steps perpendicular to the beam-path in an 8 × 8 mm^2^ area to produce a 2D cross-sectional image of the beam. Frequency-dependent spatial distribution of the broadband beam intensity at the focus was calculated using the Fourier transform of the time-domain data. The spot size can be measured directly by finding the distance between two points on the opposite sides of the maximum where the amplitude of the electric field signal fell to 1/*e*. Additionally, knife-edge measurements showed that the THz beam emitted from the PCA had a non-circular cross section producing a collimated beam with 10.6 mm and 16.2 mm diameter in the horizontal (*x*) and vertical (*y*) directions, respectively, resulting in non-circular foci for all lenses.

**FIG. 3.**
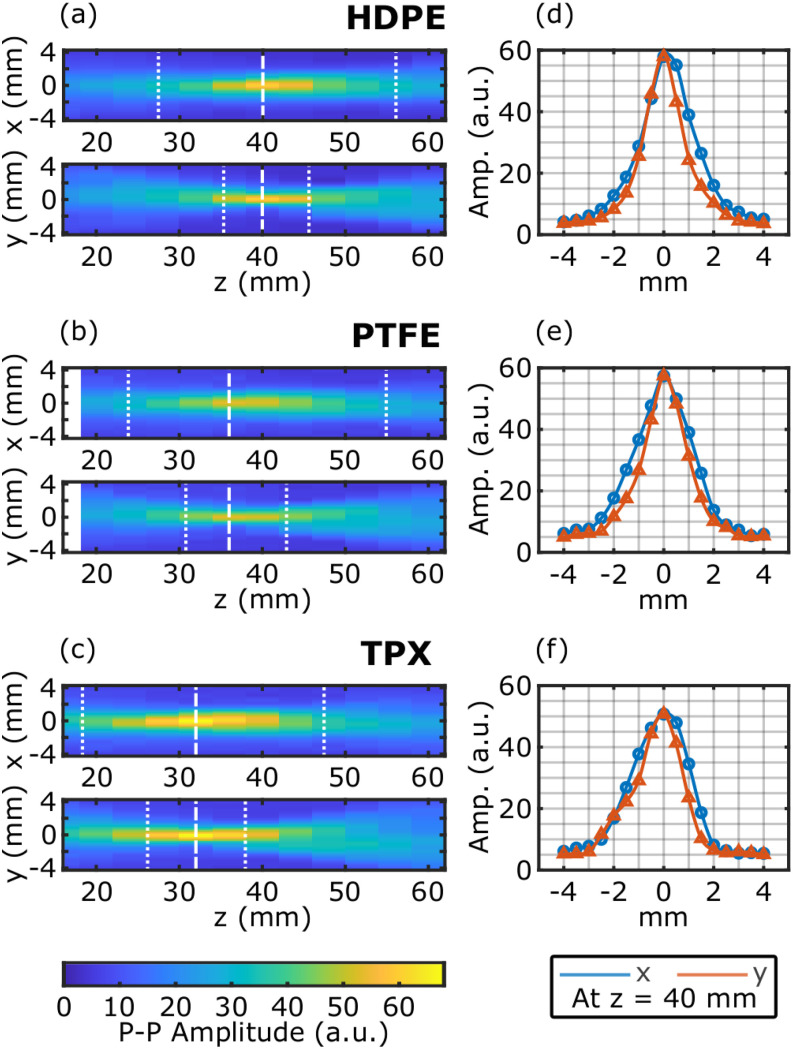
[(a)–(c)] *xz* and *yz* beam cross sections for each f-*θ* lens. The beam waist and depth of focus are marked by the dashed and dotted lines, respectively. The *z*-position is defined by the distance from the lens center plane. [(d)–(f)] The amplitude cross sections of the spot at the nominal focal plane show the Gaussian beam profile.

The depth of focus was measured by acquiring 2D images of the centered beam at several positions along the *z*-axis. Figures 3(a)–3(c) show the beam cross sections along the *xz*-direction and *yz*-direction for the three f-*θ* lenses. The depth of focus, shown by two vertical dotted lines for each direction, was calculated by finding the positions in *z*, where $D\left(\pm z_{R}\right)=D_{0}\sqrt{2}$ is the beam waist diameter. *D*_0_ = *D*(0) is the waist diameter at the focus (vertical dashed lines), and *z*_*R*_ is the Rayleigh length in either direction. Importantly, while all three f-*θ* lenses have the same nominal focal length, the observed difference in the measured positions agrees with the simulations in Fig. 1. Figures 3(d)–3(f) confirm the Gaussian profile of the THz beam at the focus. The disparity between the actual and the nominal focal length, 40 mm, especially in the case of the TPX f-*θ* lens, highlights the optimization trade-off explained earlier. As will be seen in the following discussion, the ability of the TPX lens to maintain a constant focal spot size and normal incidence angle over a large range of deflection angles comes at the cost of a shift in the focal length. The converged surface profile for the TPX f-*θ* lens here represents a local minimum in the solution to the optimization of the residue function, given the degrees of freedom in the lens design parameters. In applications where the exact focal length is more critical than the other properties of the f-*θ* lenses, the residue function in Eq. (1) can be modified accordingly.

**FIG. 4.**
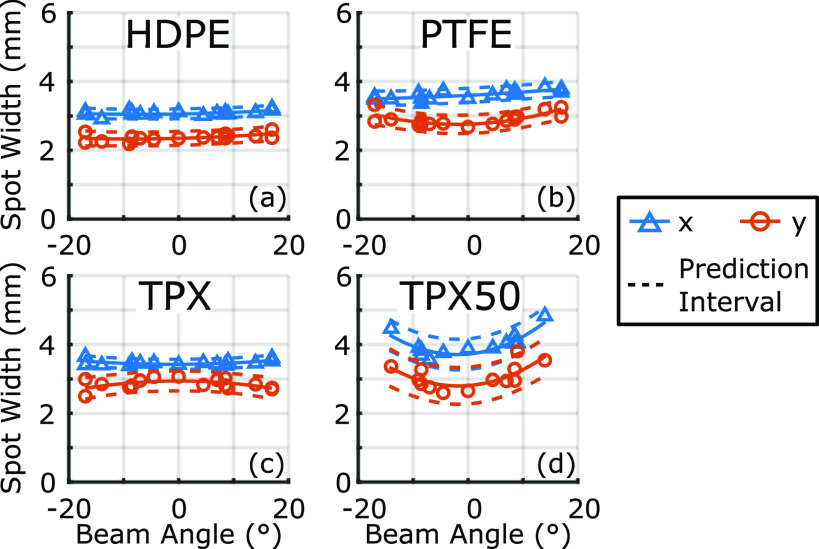
Focal spot sizes are shown as a function of beam angle using the peak-to-peak amplitude in the time domain.

**FIG. 5.**
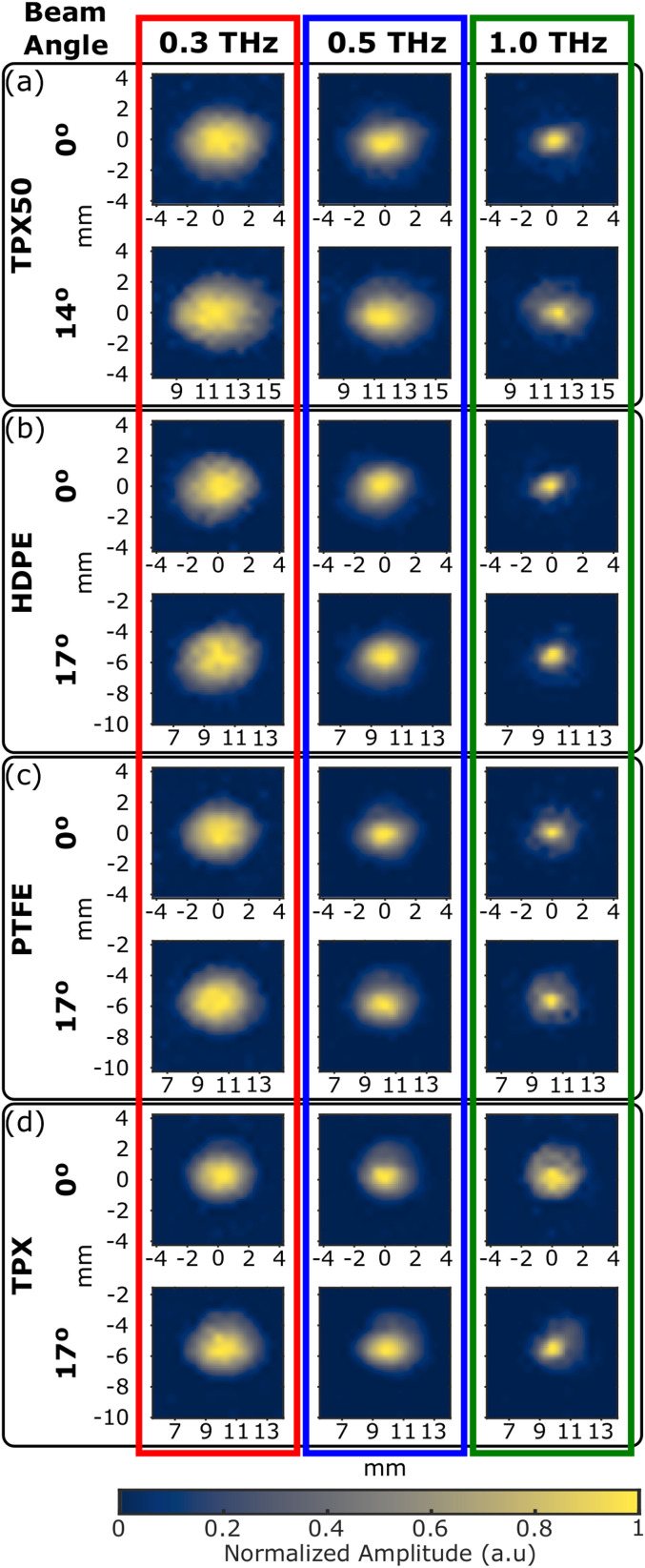
Focal spot size profiles as a function of frequency with the beam centered and steered to the maximum angle permitted by the system for (a) TPX50 and [(b)–(d)] f-*θ* lenses, each normalized by the maximum amplitude to emphasize the beam shape.

Spot size variation over the scanning field-of-view was measured by deflecting the beam and imaging the foci at the target plane. Figure 4 shows the variation in the spot width, measured by the distance between points where the peak-to-peak amplitude fell to a factor of 1/*e*, over the full range of angles achievable with our gimbal mount. In the case of the TPX50 lens, the maximum possible *θ* is smaller due to its size and focal length. Importantly, all three f-*θ* lenses maintain approximately the same spot width over the beam deflection range, while the TPX50 spot size increases despite the smaller angular range. When the foci are examined by their frequency components, a similar behavior is observed. To highlight this effect, Fig. 5 shows the shapes of the focal spot at 0.3 THz, 0.5 THz, and 1.0 THz for both a centered beam and the beam at the maximum deflection angle. Here, as well, while the focal shapes of all three f-*θ* lenses remain nearly constant between the two deflection positions, the spot size of the TPX50 lens is larger when the beam is deflected.

These observations of the spot size change agree with the focal depth variations and spot size calculations in Fig. 1 and highlight the differences between possible optimized surface profile solutions. Importantly, the property of all three f-*θ* lenses in maintaining a nearly consistent spot size over the ±17° field of view is attributed to the choice of the residue function, which uses the spot size at the target plane and is dependent on the square of the beam angle’s deviation from the normal incidence. Both of these two terms penalize any other incidence angles where the beam spot width is stretched in the plane of incidence. For the scanning lenses presented here, this consistency in response to beam deflection is the critical performance metric.

### B. Time of arrival

**FIG. 6.**
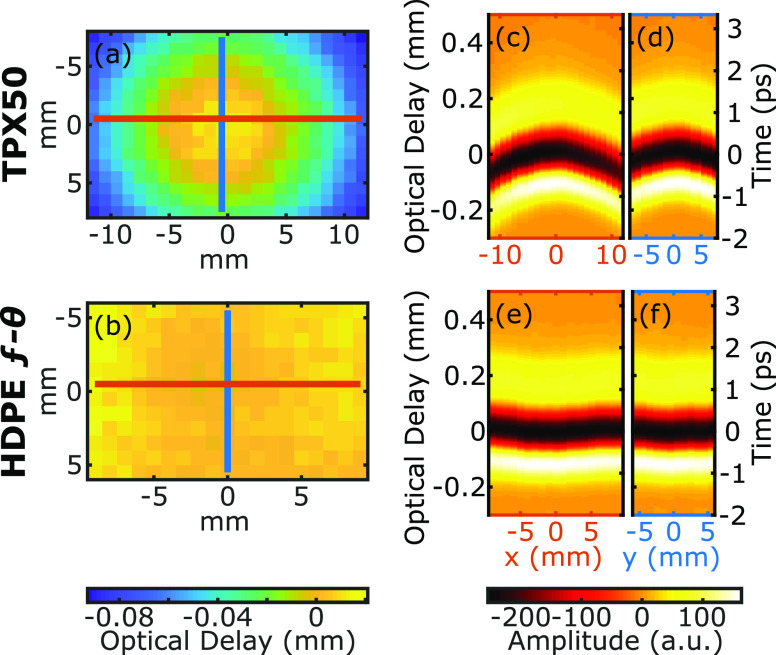
Time of arrival on a flat mirror measured using (a) the TPX50 and (b) the HDPE f-*θ* lenses. Time-domain amplitude along horizontal (red) and vertical (blue) cross-sectional lines of the TPX50 image is shown in (c) and (d), respectively. A similar cross-sectional amplitude profile for the HDPE f-*θ* lens is shown in (e) and (f).

**FIG. 7.**
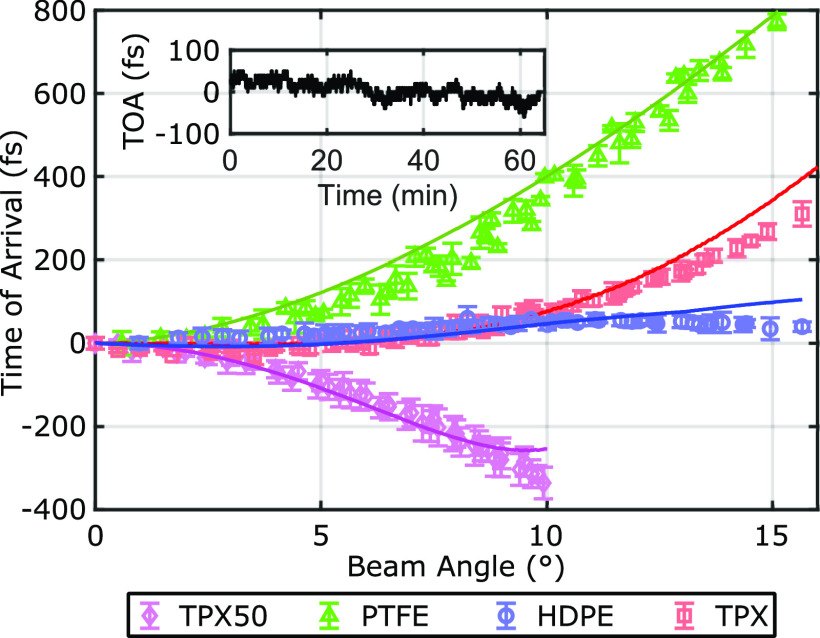
Time of arrival of the pulse for each lens. Error bars are the standard deviation of all pixels at each angle. Solid lines show the simulated TOA in excellent agreement with the data. The inset shows the laser drift measured at a constant reference position every 2.5 s over 60 min.

**FIG. 8.**
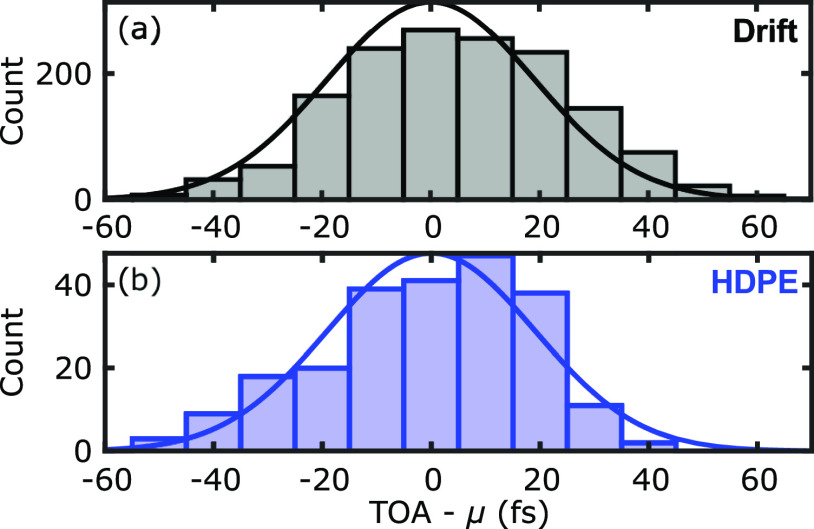
The distribution and Gaussian fit to (a) the drift measurements (*μ* = 0 fs and *σ* = 20 fs) and (b) the HDPE TOA measurements (*μ* = 39 fs and *σ* = 19 fs).

The final criterion for which the lenses were evaluated is the consistency of the time of arrival (TOA) as a function of the deflection angle. Figure 6 shows the measured optical delay for THz-TDS measurements obtained from a planar mirror imaged by the HDPE f-*θ* and TPX50 lenses. An ideal f-*θ* lens would produce constant TOA values over a flat target. This property, when combined with the consistent spot-size, would allow for the direct comparison of the phase information between two scanned positions. The cross sections in Figs. 6(b)–6(e) especially highlight the isophase behavior of the f-*θ* lens over the full transverse range. The TOA for all lenses is shown as a function of the beam deflection angle in Fig. 7 with error bars representing the standard deviations of the TOA in all pixels with the same deflection angle. The solid lines show the simulated TOA, in agreement with the experimental results. The HDPE and TPX f-*θ* lenses perform much better than the PTFE f-*θ* or TPX50 lenses in maintaining a constant TOA. The inset in Fig. 7 shows the drift in the TOA of a fixed point on a flat mirror (*θ* = 0°) during a continuous measurement over 60 min. The ∼100 fs drift is largely due to the cavity instability of the two 1560 nm femtosecond fiber lasers used in our Asynchronous Optical Sampling (ASOPS) system. In comparison to the drift measurements, the TPX and HDPE f-*θ* lenses show a close to theoretically ideal isophase performance up to 10° and 17° (the full range of the deflection angle), respectively. The poor performance of the PTFE lens in maintaining a constant TOA is especially notable, which is in agreement with previous efforts toward THz f-*θ* lenses using this material.^3,4^ Significantly, Fig. 8 demonstrates that the HDPE f-*θ* lens maintained a constant TOA with a standard deviation of 19 fs over the wide ±17° field of view. Given the <75 fs pulse duration of the femtosecond probe beam, this isophase performance is comparable to the sub-cycle time scale of the bandwidth-limited pulse in the ASOPS measurement system.

## IV. CONCLUSION

Our generalized method for the design of custom f-*θ* lenses in the THz regime was shown to yield satisfactory solutions using several polymers. However, given our design parameters, the higher index of refraction of HDPE, and its desirable smaller dispersion, the HDPE f-*θ* lens outperformed both PTFE and TPX f-*θ* lenses as well as the more conventional plano-convex TPX50 lens in transmission and reflection characterizations. Furthermore, in a telecentric temporal performance test in a reflection setup, which uses a 2-axis gimballed mirror in a telecentric beam steering configuration, the HDPE f-*θ* lens maintained a near constant phase performance over a ±17° deflection angle. This observation was limited only by the drift and pulse duration of the femtosecond fiber laser system used for the optical gating of the PCA. These results will allow for the proliferation of f-*θ* scanning lenses in the future THz imaging systems with minimized imaging distortion over a large field of view. The broadband isophase performance of the HDPE lens is uniquely suited for the full spectroscopic imaging of heterogeneous samples in reflection geometries.

## Data Availability

The data that support the findings of this study are available from the corresponding author upon reasonable request.

## References

[1] T. Araki, T. Hirai, and T. Kyotani, “Development of F-theta lens for UV lasers,” SEI Tech. Rev. 69, 59–65 (2009).

[2] J. D. Díaz, J. Stritzel, M. Rahlves, O. Majdani, E. Reithmeier, T. Ortmaier, and B. Roth, “One step geometrical calibration method for optical coherence tomography,” J. Opt. 18, 015301 (2016). 10.1088/2040-8978/18/1/015301

[3] S. Katletz, M. Pfleger, H. Pühringer, N. Vieweg, B. Scherger, B. Heinen, M. Koch, and K. Wiesauer, “Efficient terahertz en-face imaging,” Opt. Express 19, 23042–23053 (2011). 10.1364/oe.19.023042 22109184

[4] D.-S. Yee, K. H. Jin, J. S. Yahng, H.-S. Yang, C. Y. Kim, and J. C. Ye, “High-speed terahertz reflection three-dimensional imaging using beam steering,” Opt. Express 23, 5027–5034 (2015). 10.1364/oe.23.005027 25836537

[5] G. Ok, K. Park, H. S. Chun, H.-J. Chang, N. Lee, and S.-W. Choi, “High-performance sub-terahertz transmission imaging system for food inspection,” Biomed. Opt. Express 6, 1929–1941 (2015). 10.1364/boe.6.001929 26137392 PMC4467720

[6] G. Ok, K. Park, H. J. Kim, H. S. Chun, and S.-W. Choi, “High-speed terahertz imaging toward food quality inspection,” Appl. Opt. 53, 1406–1412 (2014). 10.1364/ao.53.001406 24663370

[7] S. Schumann, C. Jansen, M. Schwerdtfeger, S. Busch, O. Peters, M. Scheller, and M. Koch, “Spectrum to space transformed fast terahertz imaging,” Opt. Express 20, 19200–19205 (2012). 10.1364/oe.20.019200 23038561

[8] S. Sung, N. Bajwa, N. Fokwa, P. Tewari, R. Singh, M. Culjat, B. Nowroozi, W. Grundfest, and Z. Taylor, “Fast-scanning THz medical imaging system for clinical application,” in Terahertz Emitters, Receivers, and Applications III (International Society for Optics and Photonics, 2012), Vol. 8496, p. 84960S.

[9] Z. B. Harris, A. Virk, M. E. Khani, and M. H. Arbab, “Terahertz time-domain spectral imaging using telecentric beam steering and an f-*θ* scanning lens: Distortion compensation and determination of resolution limits,” Opt. Express 28, 26612–26622 (2020). 10.1364/oe.398706 32906931 PMC7679195

[10] A. Podzorov and G. Gallot, “Low-loss polymers for terahertz applications,” Appl. Opt. 47, 3254–3257 (2008). 10.1364/ao.47.003254 18566619

[11] B. Scherger, M. Scheller, C. Jansen, M. Koch, and K. Wiesauer, “Terahertz lenses made by compression molding of micropowders,” Appl. Opt. 50, 2256–2262 (2011). 10.1364/ao.50.002256 21614120

[12] M. Naftaly, R. Miles, and P. Greenslade, “THz transmission in polymer materials—A data library,” in *2007 Joint 32nd International Conference on Infrared and Millimeter Waves and the 15th International Conference on Terahertz Electronics* (IEEE, 2007), pp. 819–820.

[13] B. Scherger, S. Wietzke, M. Scheller, N. Vieweg, M. Wichmann, M. Koch, and K. Wiesauer, “Characterization of micro-powders for the fabrication of compression molded THz lenses,” J. Infrared, Millimeter, Terahertz Waves 32, 943–951 (2011). 10.1007/s10762-011-9806-5

[14] J. A. Nelder and R. Mead, “A simplex method for function minimization,” Comput. J. 7, 308–313 (1965). 10.1093/comjnl/7.4.308

